# Distribution of granzyme-expressing NK cells in tuberculosis reflects subset and compartment-specific remodeling

**DOI:** 10.3389/fimmu.2026.1747972

**Published:** 2026-03-17

**Authors:** Fuxiang Li, Youchao Dai, Shuixiang Xie, Rong Hu, Xueyun Gao, Xiao Huang, Shuxi Zhong, Yi Cai, Xinchun Chen, Junyun Huang

**Affiliations:** 1 Department of Laboratory Medicine, First Affiliated Hospital of Gannan Medical University, Ganzhou, China; 2 The First Clinical Medical College of Gannan Medical University, Ganzhou, China; 3 Guangzhou Eighth People’s Hospital, Guangzhou Medical University, Guangzhou, Guangdong, China; 4 School of Basic Medicine, Gannan Medical University, Ganzhou, China; 5 Department of Chemistry, Faculty of Environment and Life Science, Center of Excellence for Environmental Safety and Biological Effects, Beijing University of Technology, Beijing, China; 6 Guangdong Provincial Key Laboratory of Infection Immunity and Inflammation, Department of Pathogen Biology, Shenzhen University Medical School, Shenzhen, China

**Keywords:** CCR5, GZMA, GZMB, GZMK, natural killer cells, tuberculosis

## Abstract

**Introduction:**

Natural killer (NK) cells contribute to immunity against *Mycobacterium tuberculosis* (*Mtb*), yet their granzyme expression and subset distribution in TB remain poorly defined.

**Methods:**

NK cell subsets and the expression of granzymes (GZMA, GZMB, and GZMK) and CCR5 were analyzed by multiparametric flow cytometry in peripheral blood from healthy controls, individuals with latent TB infection, active TB patients, and treated TB patients, as well as in paired pleural fluid samples.

**Results:**

In peripheral blood from active TB patients, NK cells exhibited reduced co-expression of GZMA, GZMB, and GZMK alongside decreased subset frequencies and absolute counts, a defect restored after treatment. In contrast, pleural fluid NK cells exhibited a distinct signature characterized by elevated GZMK but reduced GZMA and GZMB. This pattern was attributable to the relative enrichment of CD56^bright^ NK cells, which are inherently high in GZMK. We also identified a CCR5^bright^ NK cell subset, phenotypically resembling CD56^bright^ NK cells with high GZMK and low GZMA/GZMB expression, that was selectively expanded in peripheral blood of TB patients and enriched in pleural effusions. This subset was inducible by in vitro *Mtb* stimulation of healthy PBMCs.

**Discussion:**

These findings reveal granzyme remodeling and altered distribution of GZMK^+^CD56^bright^ NK cells associated with CCR5^bright^ expression in TB, suggesting their potential involvement in tissue-specific NK responses.

## Introduction

Tuberculosis (TB), caused by *Mycobacterium tuberculosis* (*Mtb*), remains one of the leading global health threats, with approximately a quarter of the world’s population infected with the pathogen in a latent state (LTBI) (1). While most individuals with LTBI do not progress to active disease, 5–10% may develop TB, particularly those with weakened immune systems (2). Before adaptive immunity against *Mtb* develops, innate immune mechanisms provide the first line of defense. Natural killer (NK) cells are central to this response, they directly eliminate extracellular *Mtb* by releasing perforin, GZMB, and granulysin (3, 4). NK cells also produce cytokines and chemokines, including IFN-γ, TNF-α, IL-5\10\13, and GM-CSF, which influencing TB progression (5). Furthermore, NK cells recognize and induce apoptosis in *Mtb*-infected cells, promote immature dendritic cell maturation (6) and regulate adaptive immunity against *Mtb* by modulating CD8^+^ T cell (7, 8) and regulatory T cell expansion (9, 10). In T cell deficient mice, NK cell depletion leads to higher lung bacterial loads, more severe tissue damage, and reduced survival, highlighting their essential role in early TB defense (11).

Despite these protective functions, clinical evidence indicates that *Mtb* infection alters NK cell frequency, subset distribution, and effector molecules expression (12). Specifically, reductions in total NK cell counts and shifts in NK cell subsets have been observed (13–15), including decreases in CD56^bright^ CD16^+/–^ (16) and CD8α^+^ NK cells (17), along with diminished cytotoxicity (18–21). While these alterations are well-documented in peripheral blood, much less is known about NK cell remodeling at infection sites, such as pleural fluid. Granzymes, including GZMA, GZMB, and GZMK, are critical mediators of NK cell cytotoxicity, inducing apoptosis in infected, foreign or tumor cells (22). Recent evidence suggests that granzymes also play a direct or indirect role in suppressing *Mtb* growth (3, 23, 24), highlighting their potential role in antimicrobial defense. However, despite clear evidence of NK cell remodeling during TB, the impact of *Mtb* infection on the dynamic changes in granzyme expression during TB progression and across different anatomical compartments in TB patients remains poorly understood.

Building on previous work from our team, where we found a reduction in the percentage of peripheral blood CD3^–^CD7^+^GZMB^+^ NK subsets in advanced TB (25), and observed differential granzyme expression in CD4^+^ and CD8^+^ NK cells between peripheral blood and pleural effusions in TB patients (24), we sought to investigate the compartment- and disease state-specific remodeling of NK cell granzyme expression in TB. Specifically, we compare the granzyme expression profiles of NK cells in peripheral blood and pleural effusions from HC, LTBI, TB and RxTB. We show that, in peripheral blood, NK cells from TB patients exhibit reduced expression of GZMA, GZMB, and GZMK compared to healthy controls and latent TB, with recovery of these expressions following successful treatment. Conversely, NK cells in pleural fluid demonstrate elevated GZMK but reduced GZMA and GZMB expression, a finding linked to the presence of a CCR5^bright^ NK cell subset. Our study provides new insights into the subset- and compartment-specific remodeling of granzyme-expressing NK cells during TB.

## Results

### Depletion of peripheral GZMA^+^, GZMB^+^ and GZMK^+^ NK cells in active TB and recovery in post-therapy patients

**Figure 1:**
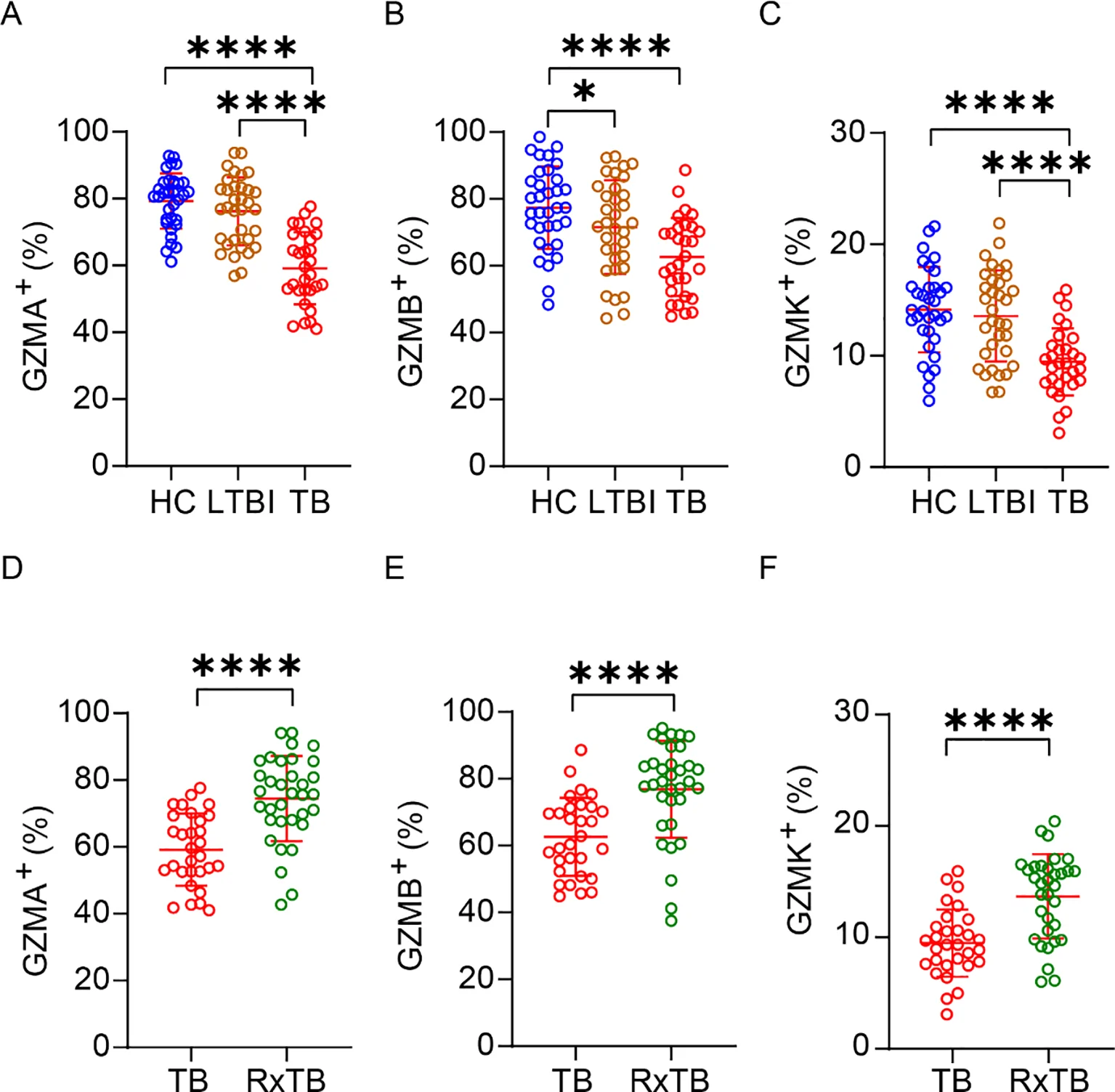
Depletion of peripheral GZMA^+^/GZMB^+^/GZMK^+^ NK cells in active TB and recovery in post-therapy patients. **(A-C)** Frequencies of peripheral blood GZMA^+^ **(A)**, GZMB^+^ **(B)**, and GZMK^+^ **(C)** NK cells in healthy controls (HC; n=34), latent TB infection (LTBI; n=34), and active TB patients (TB; n=30). **(D-F)** Frequencies of GZMA^+^ **(D)**, GZMB^+^ **(E)**, and GZMK^+^ **(F)** NK cell in active TB patients (n=30) versus post-therapy recovered individuals (RxTB, n=34). Each dot represents an individual donor. Data are shown as mean ± SEM. *P < 0.05, ****P < 0.0001 by one-way ANOVA with Tukey’s multiple comparisons test **(A–C)** or unpaired Student’s t-test **(D–F)**.

Multiple studies indicate that NK cell subset composition and key effector molecules are altered during TB progression, and our preliminary work demonstrated reduced CD3^–^CD7^+^GZMB^+^ subsets in advanced TB (25). Given that granzymes are central mediators of NK-cell cytotoxicity against *Mtb*, we investigated whether granzyme expression in NK cells is affected by TB infection. PBMCs from HC, LTBI, active TB patients, and RxTB were analyzed by flow cytometry (**Supplementary Figure 1A**). To better understand the changes in granzyme-positive NK cells during TB progression, we also quantified the absolute number of circulating NK cells. Consistent with previous reports (13–15), the total number of peripheral blood NK cells was significantly reduced in TB patients compared with HCs (**Supplementary Figure 1B**). Further analysis revealed that, compared to HCs and LTBI subjects, active TB patients exhibited significantly reduced percentages and absolute numbers of GZMA^+^, GZMB^+^, and GZMK^+^ NK cells (**Figures 1A–C**, **Supplementary Figures 1C–E**). After successful anti-TB therapy, both the total NK cell numbers and the frequencies/absolute counts of these granzyme-positive NK subsets showed significant recovery in RxTB patients. (**Figures 1D–F**, **Supplementary Figures 1F–I**). These findings demonstrate that expression of GZMA, GZMB and GZMK in peripheral NK cells is closely associated with TB disease progression.

### Broad reduction of peripheral NK cell granzyme co-expressing subsets during active TB reverses after treatment

**Figure 2:**
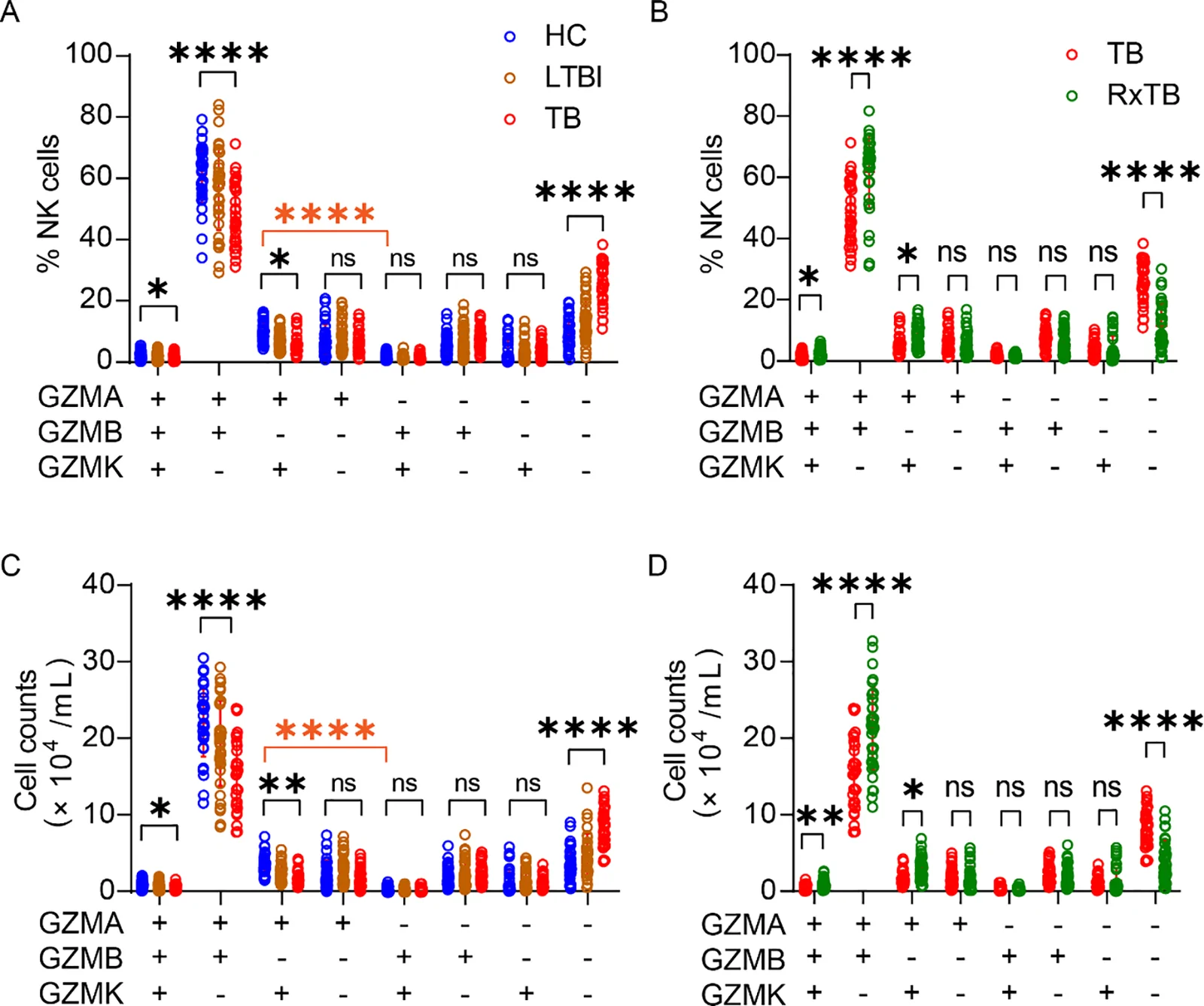
Co-expression of granzymes in peripheral blood NK cells during TB progression. **(A, B)** Frequency of triple-granzyme co-expressing NK cells was evaluated using Boolean gating in FlowJo. **(C, D)** Absolute count of triple-granzyme co-expressing NK cells, calculated from the total NK cell count and the percentage of granzyme-positive cells. Each dot represents an individual donor. Data are shown as mean ± SEM, ns, not significant, *P < 0.05, **P < 0.01, and ****P < 0.0001 by one-way ANOVA with Tukey’s multiple comparisons test **(A, C)** or unpaired Student’s t-test **(B, D)**. The orange asterisks in **(A, C)** denote significant differences between GZMA^+^GZMB^–^GZMK^+^ and GZMA^–^GZMB^+^GZMK^+^ NK cell populations in healthy controls, as assessed by unpaired Student’s t-test.

To further characterize the functional landscape of NK cells, we delineated their combinatorial granzyme expression profiles using Boolean gating analysis incorporating GZMA, GZMB, and GZMK. This analysis confirmed that the GZMA^+^GZMB^+^GZMK^–^ phenotype was predominant, whereas GZMA^+^GZMB^+^GZMK^+^ and GZMA^–^GZMB^+^GZMK^+^ populations were infrequent (**Figure 2A**). Notably, the frequencies of GZMA^+^GZMB^+^GZMK^+^, GZMA^+^GZMB^+^GZMK^–^, and GZMA^+^GZMB^–^GZMK^+^ subsets declined along with TB progression and recovered after successful treatment. In contrast, the percentage of GZMA^–^GZMB^–^GZMK^–^NK cells increased during active disease and decreased post-therapy (**Figures 2A, B**). NK cells expressing only a single granzyme (GZMA, GZMB, or GZMK alone) did not show significant changes across disease states (**Figures 2A, B**). We further quantified the absolute numbers of these granzyme-defined subsets. Consistent with the frequency data, the absolute counts of GZMA^+^GZMB^+^GZMK^+^, GZMA^+^GZMB^+^GZMK^–^, and GZMA^+^GZMB^–^GZMK^+^ NK cells decreased during active TB and restored after treatment (**Figures 2C, D**), whereas GZMA^-^GZMB^-^GZMK^-^ NK cells showed the opposite trend. Given that human NK cells express multiple other granzymes, it remains unclear whether the expanded GZMA^–^GZMB^–^GZMK^–^ subset in TB expresses other granzyme family members, which warrants further investigation.

These results indicate that GZMA^+^GZMB^+^GZMK^–^ represents the major NK cell subset, highlighting a prevalent co-expression pattern of GZMA and GZMB. In contrast, most GZMK^+^ NK cells co-expressed GZMA but not GZMB (**Figures 2A, C**), forming distinct GZMK^+^GZMA^+^ and GZMK^+^GZMB^–^ clusters (**Supplementary Figure 2**). Collectively, these data demonstrate that the progression of active TB is associated with a broad and coordinated reduction in the frequencies and absolute numbers of nearly all granzyme-co-expressing NK cell subsets, which is reversed after treatment. This pattern indicates a generalized impairment of NK cell effector function during active disease, extending beyond the loss of single granzyme-expressing cells.

### Peripheral blood and pleural fluid NK cells from TB patients exhibit differing granzyme expression profiles

**Figure 3:**
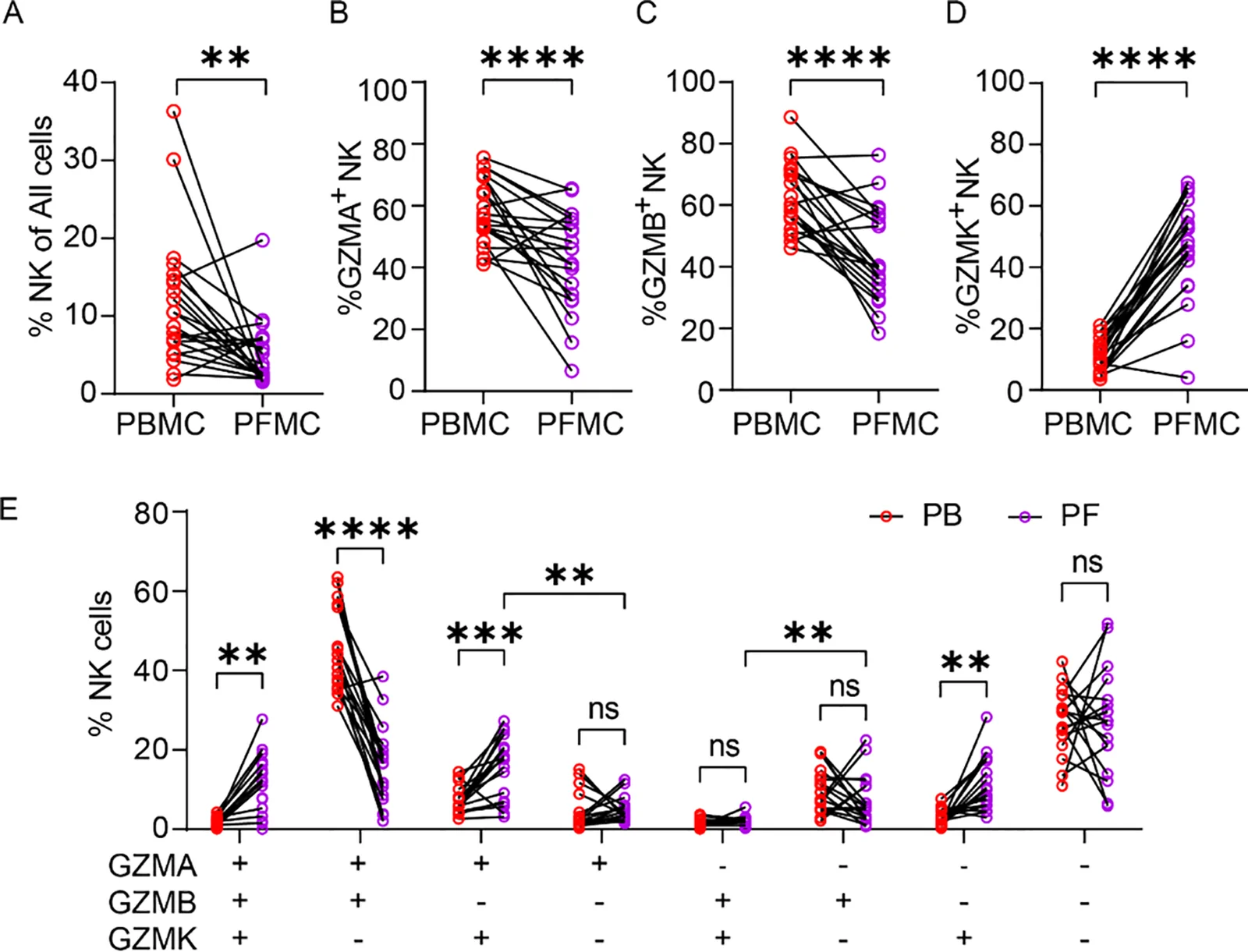
Compartmental differences in granzyme expression between PFMC- and PBMC-derived NK cells. **(A)** Frequency of total NK cells in paired PBMCs and PFMCs samples from TPE patients (n=21). **(B-D)** Paired comparison of the proportions of GZMA^+^ **(B)**, GZMB^+^ **(C)**, and GZMK^+^ **(D)** NK cells in PBMC versus matched PFMC. **(E)** Co-expression analysis of GZMA/GZMB/GZMK phenotype in NK cells from PBMC and PFMC compartments. Data are shown as means ± SEM, ns, not significant, **P < 0.01, ***P < 0.001, and ****P < 0.0001 by paired Student’s t-test **(A-E)**. Specific comparisons between GZMA^+^GZMB^-^GZMK^+^ vs GZMA^+^GZMB^-^GZMK^-^, and GZMA^-^GZMB^+^GZMK^+^ vs GZMA^-^GZMB^+^GZMK^-^ subsets were analyzed using unpaired Student’s t-test.

Tuberculous pleural effusions (TPE), resulting from delayed hypersensitivity to subpleural TB lesions, are enriched in immune cells heavily exposed to *Mtb* antigens (26). To examine how disease-site NK cells differ from peripheral NK cells, we analyzed paired PBMCs and PFMCs from TB patients (gating strategy shown in **Supplementary Figures 3A, B**). Flow cytometry analysis revealed a significantly lower frequencies of NK cells in PFMCs compared to PBMCs (**Figure 3A**). Furthermore, the NK cells in PFMCs exhibited reduced proportions of GZMA^+^ and GZMB^+^ subsets. Quantitatively, the mean frequency of GZMA^+^ NK cells decreased from 58.0% in PBMCs to 42.5% in PFMCs, corresponding to an average reduction of 15.5 ± 4.2% (**Figure 3B**). A slightly greater reduction was observed for GZMB^+^ NK cells, whose mean frequency declined from 62.8% to 43.9%, representing a decrease of 18.9 ± 4.1% (**Figure 3C**). In contrast, the GZMK^+^ subsets was markedly enriched in PFMCs, with the mean frequency increasing from 11.5% in PBMCs to 45.9% in PFMCs, corresponding to an average increase of 34.4 ± 3.8% (**Figure 3D**). Granzyme co-expression profiling further demonstrated that the proportions of GZMA^+^GZMB^+^GZMK^+^, GZMA^+^GZMB^–^GZMK^+^, and GZMA^–^GZMB^–^GZMK^+^ NK subsets were markedly elevated in PFMCs, whereas the GZMA^+^GZMB^+^GZMK^–^ subset was significantly reduced (**Figure 3E**). To explore mechanisms underlying GZMK^+^ NK enrichment, we stimulated healthy donor PBMCs with *Mtb* strains (H37Ra or H37Rv), TGF-β (an NK activator abundant in TPE), or TB pleural fluid supernatant (PFS), either alone or combined with H37Rv. None of these stimuli significantly altered the frequency of GZMK^+^ NK cells (**Supplementary Figures 3C–E**). These results indicate that the distinct granzyme expression profiles in NK cells from the peripheral blood and pleural cavity of TB patients are not solely attributable to direct stimulation by *Mtb*, but likely reflect broader compartment-specific regulatory mechanisms.

### Increased GZMK^+^ NK cells in pleural fluid arise from CD56^bright^ NK cell enrichment

**Figure 4:**
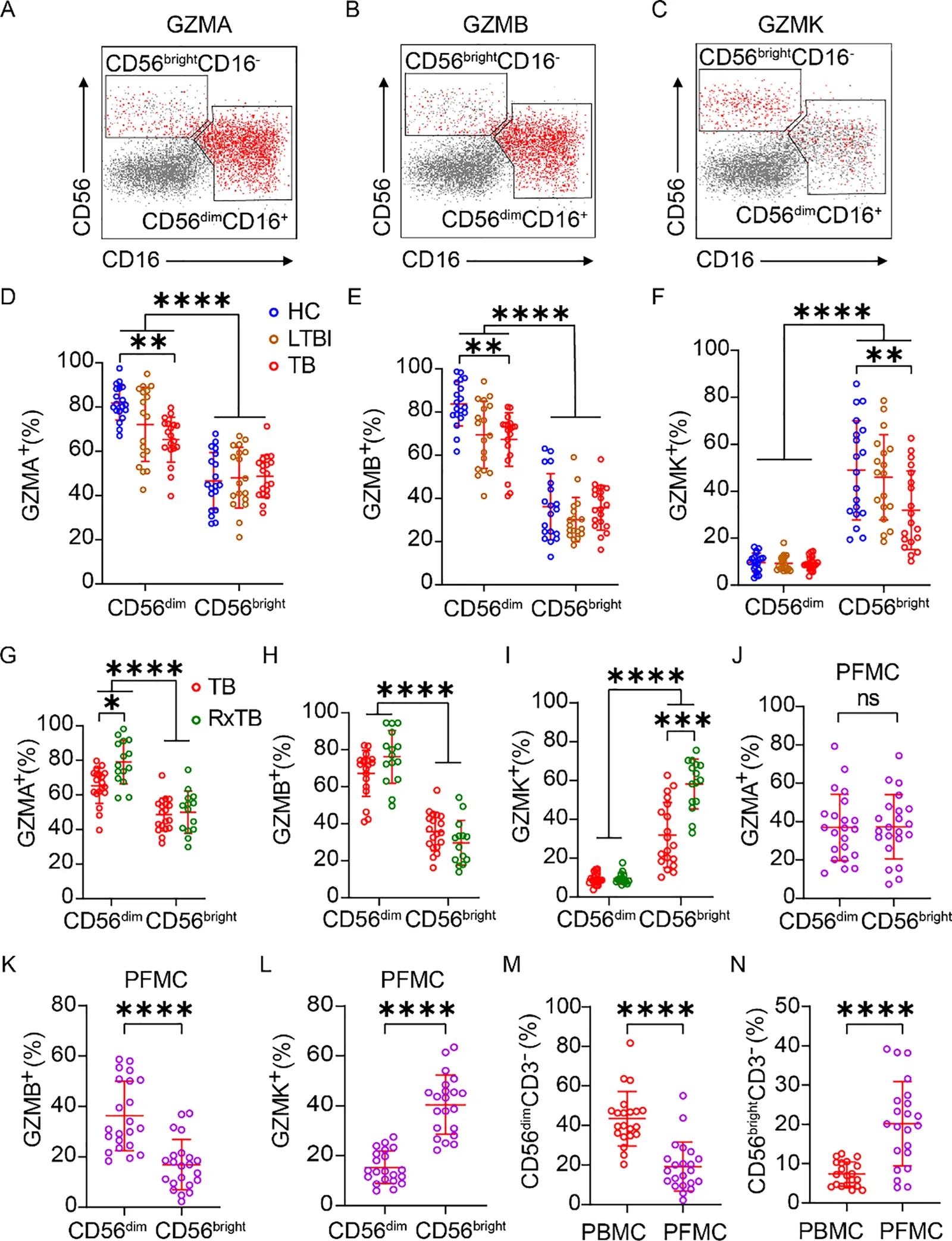
Increased GZMK^+^ NK cells in pleural fluid Arise from CD56^bright^ NK cell enrichment. **(A-G)** Representative biaxial flow cytometry plots of CD56 versus CD16 showing granzyme^+^ (red) and granzyme^–^ (gray) NK cells. Panels illustrate GZMA **(A)**, GZMB **(B)** and GZMK **(C)** distributions. **(D-I)** Frequency of GZMA^+^, GZMB^+^, and GZMK^+^ cells within the CD56^bight^ and CD56^dim^ NK subsets in PBMCs samples from HC, LTBI, TB and RxTB patient. **(J-L)** Frequency of GZMA^+^, GZMB^+^, and GZMK^+^ cells within the CD56^bight^ and CD56^dim^ subsets in PFMCs samples from TPE patient. **(M, N)** Comparison of overall CD56^bight^ and CD56^dim^ NK cell frequencies between matched PBMCs and PFMCs. Each dot represents one donor. Data are shown as means ± SEM, ns, not significant, *P < 0.05, **P < 0.01, ***P < 0.001, and ****P < 0.0001. Comparisons between CD56^bright^ and CD56^dim^ subsets within the same clinical group were analyzed by two-way ANOVA with Tukey’s multiple comparisons test **(D-I)**. Comparisons across clinical groups within the CD56^bright^ or CD56^dim^ subsets were analyzed by one-way ANOVA with Tukey’s multiple comparisons test **(D-I)**. Unpaired Student’s t-test was used for paired-compartment analyses **(J-N)**.

Human NK cells are classified into CD56^bright^ and CD56^dim^ subsets, with CD56^bright^ NK cells lacking CD16 (FcγRIIIa), exhibiting low cytotoxicity, and secreting high levels of cytokines, whereas CD56^dim^ NK cells express CD16, demonstrate potent cytotoxicity, and produce fewer cytokines (27, 28). Given this established dichotomy, we further analyzed their granzyme expression in PBMC and PFMC compartments, as detailed in the gating strategy (**Supplementary Figure 4**). We observed distinct distribution patterns of GZMA, GZMB, and GZMK between CD56^bright^ and CD56^dim^ subsets (**Figures 4A–C**). In PBMCs, GZMA and GZMB were predominantly expressed in CD56^dim^ NK cells and were significantly downregulated during active TB. In contrast, expression of these granzymes was minimal in CD56^bright^ NK cells and remained unaffected by TB status (**Figures 4D, E**). Conversely, GZMK expression was primarily restricted to CD56^bright^ NK cells and decreased significantly during TB, while remaining low and stable in CD56^dim^ subset (**Figure 4F**). Following treatment, the frequencies of GZMA^+^ CD56^dim^ and GZMK^+^ CD56^bright^ cells significantly rebounded, and GZMB^+^ CD56^dim^ cells also showed a trending increase (**Figures 4G–I**). In PFMCs, the proportions of GZMA^+^ cells were uniformly low across CD56^dim^ and CD56^bright^ subsets without inter-subset differences (**Figure 4J**). GZMB expression remained preferentially associated with CD56^dim^ NK cells, similar to the pattern in PBMCs (**Figure 4K**), while GZMK expression remained highest in CD56^bright^ subsets (**Figure 4L**), mirroring PBMC distribution. Importantly, PFMCs contained a significantly lower proportion of CD56^dim^ NK cells and a correspondingly higher frequency of CD56^bright^ NK cell compared to matched PBMCs (**Figures 4M, N**), consistent with prior reports (29). Collectively, these findings suggest that the higher frequency of GZMK^+^ NK cells in PFMCs compared with PBMCs is attributable to the enrichment of CD56^bright^ NK cells, which are the primary GZMK-expressing subset.

### Granzyme expression patterns differ between CD56^bright^ and CD56^dim^ NK subsets and exhibit distinct changes during TB progression

**Figure 5:**
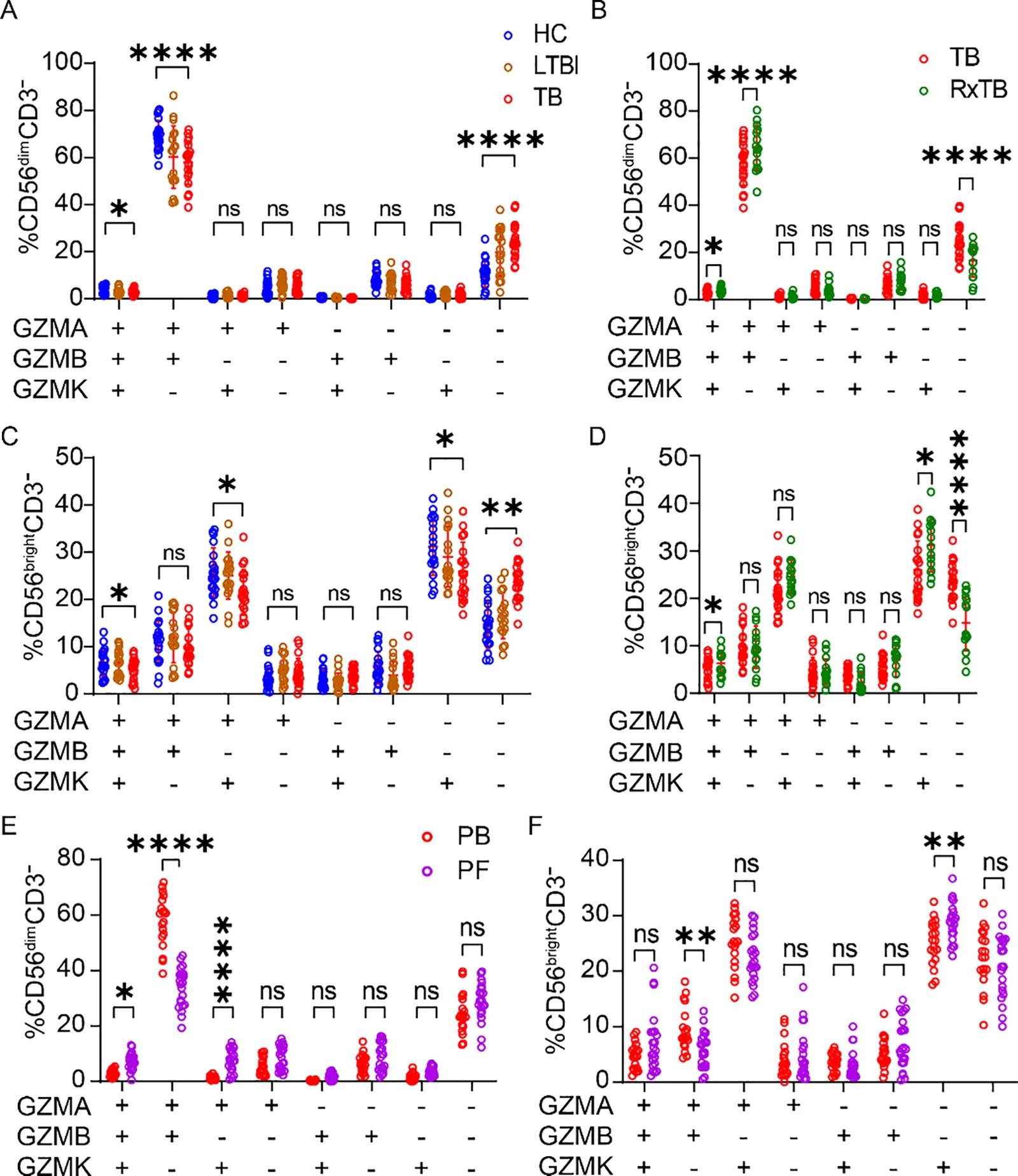
Granzyme co-expression within CD56^dim^ and CD56^bright^ NK cell subsets. **(A, B)** Frequency of triple-granzyme co-expressing cells within the CD56^dim^ NK cell subset from PBMCs of different donor groups (HC, LTBI, TB in **A**; TB and RxTB in **B**). **(C, D)** Frequency of triple-granzyme co-expressing cells within the CD56^bright^ NK cell subset from PBMCs of different donor groups (HC, LTBI, TB in **C**; TB and RxTB in **D**). **(E, F)** Comparison of triple-granzyme co-expressing cells between PBMCs and PFMCs in TB patients, within CD56^dim^ **(E)** and CD56^bright^ **(F)** subsets. Each dot represents an individual donor. Data are shown as mean ± SEM, ns, not significant, *P < 0.05, **P < 0.01, and ****P < 0.0001 by one-way ANOVA with Tukey’s multiple comparisons test **(A, C)** or unpaired Student’s t-test **(B, D–F)**.

To delineate subset-specific profiles, we separately analyzed the co-expression patterns of GZMA, GZMB, and GZMK within CD56^dim^ and CD56^bright^ NK cells. Within the CD56^dim^ subset, the GZMA^+^GZMB^+^GZMK^-^ phenotype was the predominant population, accounting for a significantly higher proportion than all other subsets (**Figure 5A**). Notably, among CD56^dim^ compartment, both the frequencies and absolute numbers of GZMA^+^GZMB^+^GZMK^+^ and GZMA^+^GZMB^+^GZMK^-^ NK cells decreased during active TB and recovered post-treatment (**Figures 5A, B**, **Supplementary Figures 5A, B**). In contrast to the pattern observed in total NK cells, the GZMA^+^GZMB^-^GZMK^+^ population within CD56^dim^ cells did not change significantly during disease progression and remained low in frequency (**Figures 2A**, **5A**). Mirroring the trend in total NK cells, the percentage and absolute number of GZMA^–^GZMB^–^GZMK^–^ CD56^dim^ NK cells increased during active TB and decreased after therapy (**Figures 2**, **5A, B**, **Supplementary Figures 5A, B**). Additionally, although the frequency of the GZMA^–^GZMB^+^GZMK^–^subset was unaltered by disease state, its absolute count was reduced in TB patients (**Supplementary Figure 5A**).

In contrast to CD56^dim^ NK cells, CD56^bright^ NK cells displayed a distinct granzyme co-expression profile and disease-associated pattern. The dominant subsets within CD56^bright^ NK cells were GZMA^+^GZMB^-^GZMK^+^ and GZMA^-^GZMB^-^GZMK^+^, both of which showed marked reductions during TB progression and significant recovery after treatment (**Figures 5C, D**). The GZMA^+^GZMB^+^GZMK^-^ subset was present at low frequencies in CD56^bright^ NK cells and did not exhibit the TB-associated changes observed in total NK cells (**Figures 2A**, **5C**). Although the frequency of GZMA^-^GZMB^-^GZMK^-^ CD56^bright^ NK cells increased during active TB and declined after treatment, their absolute numbers did not change significantly across disease stages (**Figures 5C, D**, **Supplementary Figures 5C, D**). Importantly, the absolute counts of several CD56^bright^ NK cell subsets, including GZMA^+^GZMB^+^GZMK^+^, GZMA^+^GZMB^+^GZMK^-^, GZMA^+^GZMB^-^GZMK^+^, GZMA^-^GZMB^+^GZMK^-^, and GZMA^-^GZMB^-^GZMK^+^ were decreased during active TB (**Supplementary Figure 5C**), providing further evidence that CD56^bright^ NK cells are reduced in peripheral blood over the course of disease progression.

Comparative analysis of granzyme expression in CD56^dim^ and CD56^bright^ subsets between PBMCs and PFMCs revealed further distinctions. In CD56^dim^ NK cells, the GZMA^+^GZMB^+^GZMK^+^ and GZMA^+^GZMB^-^GZMK^+^ subsets were significantly enriched in PFMCs, whereas the predominant GZMA^+^GZMB^+^GZMK^-^ subset was markedly reduced, consistent with the distribution observed in total NK cells (**Figures 3E**, **5E**). In contrast, CD56^bright^ NK cells showed no significant differences in the frequencies of GZMA^+^GZMB^+^GZMK^+^ or GZMA^+^GZMB^-^GZMK^+^ subsets between PBMCs and PFMCs. However, the GZMA^-^GZMB^-^GZMK^+^ subset was significantly enriched in PFMCs (**Figure 5F**), further supporting the notion that the increased abundance of GZMK^+^ NK cells in pleural fluid is primarily driven by the enrichment of CD56^bright^ NK cells at the site of infection.

### CCR5^bright^-associated redistribution of GZMK^+^CD56^bright^ NK cells from circulation to the pleural space in active tuberculosis

**Figure 6:**
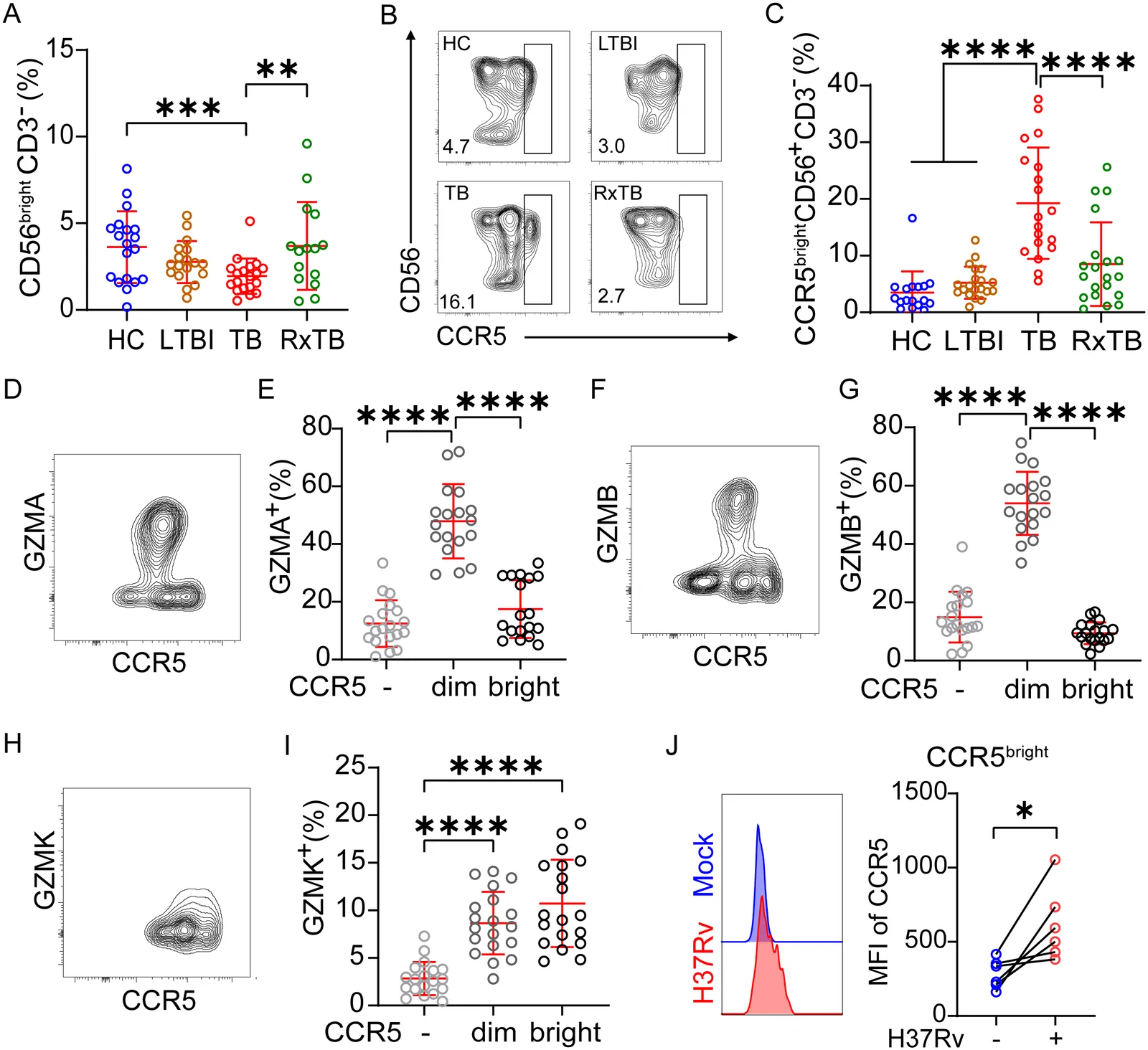
CCR5^bright^ NK cells are preferentially GZMK^+^ and increase during active TB. **(A)** Frequencies of CD56^bright^ NK cell subsets in PBMCs from HCs, LTBI, TB, and RxTB. **(B)** Contour plots showing CD56 versus CCR5 expression on CD3^–^ cells from different donor groups. CD56^+^CCR5^+^ cells in TB patients can be classified into three distinct subsets: CCR5^–^, CCR5^dim^, and CCR5^bright^. **(C)** Percentages of CCR5^bright^ cells among CD56^+^CD3^–^ cells. The CCR5^bright^ subset was significantly increased in active TB patients compared to HCs, LTBI individuals, and RxTB patients. **(D, F, H)** Contour plots illustrating granzyme expression versus CCR5 in NK cells from TB patients. **(E, G, I)** Proportions of GZMA^+^, GZMB^+^ and GZMK^+^ NK cells within CCR5-defined subsets. GZMK^+^ NK cells were preferentially enriched in the CCR5^bright^ population, whereas GZMA^+^ and GZMB^+^ distributions showed no such bias. **(J)** Following *ex vivo* H37Rv infection (MOI = 5, 24 h), mean fluorescence intensity (MFI) of CCR5 increased significantly on CD56^bright^ NK cells. Data are shown as means ± SEM, ns, not significant, *P < 0.05, **P < 0.01, ***P < 0.001, and ****P < 0.0001 by one-way ANOVA with Tukey’s multiple comparisons test **(A, C, E, G, I)** or unpaired Student’s t-test **(J)**.

To investigate the mechanistic basis for increased CD56^bright^ NK cell frequencies in PFMCs relative to PBMCs, we first examined peripheral CD56 subset dynamics across clinical stages. The proportions and absolute count of CD56^bright^ NK cell in peripheral blood were reduced in active TB but returned to baseline after treatment (**Figure 6A**, **Supplementary Figure 6A**), whereas CD56^dim^ frequencies and absolute counts showed no significant change (**Supplementary Figures 6B, C**). We therefore hypothesized that CD56^bright^ NK cells in peripheral blood migrate into the pleural compartment during disease progression. Previous studies have identified a CCR5^bright^ CD4^+^ effector memory cells that exhibits exceptionally high GZMK expression (30). CCR5 interacts with multiple CC chemokines, including CCL2, CCL3, CCL7 and CCL8 (31), which are significantly enriched in the pleural fluid of TB patients (32–34). On this basis, we asked whether CCR5 mediate GZMK^+^NK-cell subset migration.

Contour plots of CD56 versus CCR5 revealed two distinct subsets among CD56^+^ cells in HC, LTBI, and RxTB groups, whereas TB patients exhibited three subsets based on CCR5 expression levels (**Figure 6B**, **Supplementary Figure 6D**), which we designated as CCR5^–^, CCR5^dim^, and CCR5^bright^, paralleling previous observations in CCR5^bright^ CD4^+^ effector memory T cells (30). Using the gating strategy outlined in **Supplementary Figure 6D**, we analyzed the frequency and absolute count of CCR5^bright^ cells within CD3^–^CD56^+^ NK cells. Relative to HCs and LTBI subjects, the CCR5^bright^ NK subset exhibited a significant increase in both percentage and absolute number in active TB patients, which substantially declined after treatment (**Figure 6C**, **Supplementary Figure 6E**), suggesting that this increase was not attributable to a contraction of other NK subpopulations. We further evaluated granzyme expression profiles across these CCR5-defined NK subpopulations in TB patients. GZMA and GZMB were predominantly expressed in CCR5^dim^ NK cells and were significantly lower in both CCR5^–^ and CCR5^bright^ subsets (**Figures 6D–G**). In contrast, GZMK was highly expressed in both CCR5^dim^ and CCR5^bright^ NK cells (**Figures 6H, I**). Additionally, *In vitro* H37Rv stimulation significantly augmented CCR5 surface density (mean fluorescence intensity, MFI) specifically on CD56^bright^ NK cells derived from healthy donor PBMCs (**Figure 6J**), with no significant effect observed on the CD56^dim^ subset (**Supplementary Figure 6F**). Together, these data indicate that TB progression selectively expands a CCR5^bright^ NK subset with high GZMK but low GZMA/GZMB. This specific phenotype aligns with pleural fluid enrichment profiles, supporting a CCR5-dependent trafficking model for GZMK^+^CD56^bright^ NK cells from circulation to the pleural space.

## Discussion

Clinical and single-cell studies consistently report altered NK cell frequency and function during TB progression, with partial recovery following treatment (12, 21, 35). Our data extend these observations by demonstrating a coordinated downregulation in both the frequency and absolute numbers of NK cells co-expressing GZMA, GZMB and GZMK in PBMC-derived NK cells from active TB patients, with partial or full recovery after therapy. Moreover, we reveal a compartment-specific remodeling of granzyme expression: pleural fluid NK cells exhibited significantly elevated GZMK but diminished GZMA and GZMB compared to their blood counterparts. The enrichment of GZMK^+^CD56^bright^ NK cells in pleural effusions was further associated with the expansion of a distinct CCR5^bright^ NK population in TB patients.

Natural killer (NK) cells are rapid, non-MHC-restricted effectors of innate immunity and play a critical role in early host defense against *Mtb* (22). Previous studies have reported a decline in NK cell numbers during the transition from latent infection to active disease, with recovery following treatment (15). Single-cell sequencing has further identified selective depletion of peripheral CD7^+^GZMB^+^CD3^–^ cells (mainly NK cells) in active TB, distinguishing active TB from LTBI and healthy controls (25). Additionally, circulating NK cells from TB patients exhibit impaired IFN-γ production, which improves after therapy (18, 20, 36). Our data extend these findings by revealing that NK cell dysfunction in active TB is characterized not only by numerical changes but also by a profound functional impairment, evidenced by a broad reduction in granzyme-co-expressing subsets at both the frequency and absolute count levels.

Importantly, our subset analysis reveals that granzyme expression patterns differ fundamentally between CD56^dim^ and CD56^bright^ NK subsets and exhibit distinct changes during TB progression. The loss of cytotoxic potential during active disease was primarily driven by changes within the CD56^dim^ subset, including the reduction of major co-expressing populations like GZMA^+^GZMB^+^GZMK^–^. In contrast, the CD56^bright^ subset, which naturally expresses high GZMK but low GZMA/GZMB, showed a different pattern of change. This subset-specific granularity clarifies the overall granzyme landscape and underscores that TB-associated NK cell dysfunction is not uniform across all subsets.

NK cells reside in various tissues, where they contribute to organ-specific immune responses (37–39). In the case of TB, the lungs serve as the primary site of infection and a gateway for extrapulmonary dissemination (40). However, research on PFMC-derived NK cells in TB remain scarce. Consistent with previous work (29), Our paired analysis found that PFMCs contain fewer NK cells overall, a higher proportion of CD56^bright^ CD16^–^ NK cells, and a lower proportion of CD56^dim^ CD16^+^ NK cells than matched PBMCs. Consequently, GZMK^+^ NK cells are enriched in PFMCs, reflecting the predominance of the CD56^bright^ subset, while GZMA^+^ and GZMB^+^ NK cells are diminished, aligning with their association with the CD56^dim^ subset. Chemokine-driven recruitment plays a critical role in the effective immune response against *Mtb*. In mice infected with *Mtb*, CCR5-expressing cells, including lymphocytes and macrophages, infiltrate the lungs (41). CCR5 interacts with multiple ligands, such as CCL2, CCL3, CCL5, CCL7, and CCL8 (31). In TB patients, monocytes derived from lymph node tissues and alveolar macrophages produce higher levels of CCL2, CCL3, and CCL5 than those from peripheral blood (42). Similarly, tuberculous pleural effusions contain significantly elevated levels of CCL2, CCL3, and CCL7 compared to effusions caused by other diseases (32, 33). CCL8 is also markedly higher in TB pleural fluid than in peripheral blood (34). Thus, we speculate that CCR5 may mediate the recruitment of CD56^bright^ NK cells into the pleural space in response to these chemokines. Our study identifies an increased frequency of a CCR5^bright^ NK cells in peripheral blood from active TB patients. The CCR5^bright^ subset is enriched for GZMK but not GZMA or GZMB, echoing observations in CCR5^bright^ CD4^+^ effector memory T cells (30). *In vitro* infection with H37Rv selectively increased CCR5 expression on CD56^bright^ NK cells without affecting the CD56^dim^ subset. This suggests that CCR5^bright^ NK cells, enriched for GZMK, may migrate from the circulation into pleural space, where their peripheral fraction is reduced in active TB.

In summary, while previous studies have documented NK-cell depletion in TB, our research highlights a complementary functional deficit in NK cells, specifically a reduction in granzyme-expressing subsets. We provide the first comprehensive characterization of granzyme profiles in pleural NK cells, linking their distinct pattern to the differential distribution of CD56 subsets. Furthermore, we identify an expansion of CCR5^bright^ NK cells in active TB, enriched for GZMK and associated with compartmental migration. These findings suggest that granzyme remodeling and CCR5-mediated trafficking are key mechanisms reshaping NK cell function and distribution in TB.

## Methods

### Ethics statement

This study received approval from the Ethics Committee of Shenzhen Third People’s Hospital, and Board of the Shenzhen University School of Medicine, China. All participants provided written informed consent. Experimental procedures strictly adhered to institutional ethical guidelines and biosafety protocols.

### Participants

Peripheral blood samples were collected from HC (n = 34), LTBI (n = 34), and patients with active TB (n = 30) admitted to Shenzhen Third People’s Hospital (China) between August 2021 and March 2024. None of the active TB patients had a prior history of TB or anti-TB treatment at the time of enrollment. An additional cohort of patients who had received 1 to 10 months of RxTB (n = 34) was also recruited. Active TB was diagnosed based on clinical symptoms, chest radiography, positive sputum smear or culture for *Mtb*, and clinical response to anti-TB therapy. A validated IFN-γ ELISPOT assay specific for *Mtb* (IGRA) was used to distinguish LTBI cases from uninfected HCs (25). Additionally, paired PBMCs and PFMCs were obtained from 21 patients with TPE for flow cytometric analysis and NK cell isolation. TPE was confirmed by the presence of exudative pleural fluid with culture-positive results for *Mtb* (from pleural fluid, pleural biopsy, or sputum), and/or by histological evidence of granulomatous inflammation with acid-fast bacilli on pleural biopsy.

### Isolation and processing of PBMCs and PFMCs

Heparinized peripheral blood was collected via venipuncture from all participants. pleural fluid was obtained from patients diagnosed with TPE. PBMCs were isolated by density gradient centrifugation using Ficoll-Paque Plus (Amersham Biosciences). PFMCs and PFS were collected by centrifuging up to 50 mL of pleural fluid at 300 × g for 5 minutes at 4 °C. Freshly isolated PBMCs and PFMCs were either immediately processed for flow cytometry or used for NK cell isolation via magnetic separation. PFS were stored at -80 °C for subsequent analyses. For *in vitro* stimulation assays, PBMCs from HCs were incubated for 24 hours with H37Rv, H37Ra, TGF-β, 20% PFS, alone or in combination (H37Rv + TGF-β, H37Rv + 20% PFS), according to the experimental design. After stimulation, cells were harvested for flow cytometric analysis as described below.

### Flow cytometry and intracellular cytokine staining

PBMCs and PFMCs from TB patients were stained for surface and intracellular markers using standard protocols (24). Surface staining was performed using monoclonal antibodies against CD3 (clone SK-7, BioLegend #344816), CD16 (3G8, BioLegend #302008), and CD56 (5.1H11, BioLegend #9362546), along with Ghost Dye (BioLegend #423102) for viability exclusion. Cells were incubated with antibodies for 30 minutes at 4 °C. After fixation and permeabilization (50 minutes at 4 °C), intracellular staining was performed using antibodies against granzyme A (GZMA; CB9, BioLegend #507215), granzyme B (GZMB; QA18A28, BioLegend #396414), and granzyme K (GZMK; GM26E7, BioLegend #370510). CCR5 was detected with HM-CCR5 (BioLegend, catalog #107018). All antibodies were validated by the manufacturer for flow cytometric applications. Stained cells were resuspended in 200 μL of 2% paraformaldehyde, acquired using a BD FACSAria™ III flow cytometer with FACSDiva software (BD Biosciences), and analyzed with FlowJo v10. Granzyme co-expression was assessed using Boolean gating in FlowJo v10.

### Statistical analysis

Sample size calculations and statistical analyses were performed using GraphPad Prism 8. Depending on their distribution, differences between two groups were analyzed using either the independent sample t-test or the Mann-Whitney U test. For multiple group comparisons, the ANOVA or Kruskal-Wallis test was applied, contingent on data normality. All hypothesis tests were two-sided, and a *p*-value of less than 0.05 was considered statistically significant.

## Data Availability

The original contributions presented in the study are included in the article/**Supplementary Material**. Further inquiries can be directed to the corresponding authors.
