# Correction: Distribution of granzyme-expressing NK cells in tuberculosis reflects subset and compartment-specific remodeling

**DOI:** 10.3389/fimmu.2026.2005817

**Published:** 2026-10-09

**Authors:** Fuxiang Li, Youchao Dai, Shuixiang Xie, Rong Hu, Xueyun Gao, Xiao Huang, Shuxi Zhong, Yi Cai, Xinchun Chen, Junyun Huang

**Affiliations:** 1 Department of Laboratory Medicine, First Affiliated Hospital of Gannan Medical University, Ganzhou, China; 2 The First Clinical Medical College of Gannan Medical University, Ganzhou, China; 3 Guangzhou Eighth People’s Hospital, Guangzhou Medical University, Guangzhou, Guangdong, China; 4 School of Basic Medicine, Gannan Medical University, Ganzhou, China; 5 Department of Chemistry, Faculty of Environment and Life Science, Center of Excellence for Environmental Safety and Biological Effects, Beijing University of Technology, Beijing, China; 6 Guangdong Provincial Key Laboratory of Infection Immunity and Inflammation, Department of Pathogen Biology, Shenzhen University Medical School, Shenzhen, China

**Keywords:** CCR5, GZMA, GZMB, GZMK, natural killer cells, tuberculosis

In the published article, there was an error in the **Funding** statement. “ … This work was supported by grants from the Shenzhen Medical Research Funding Program (Nos. B2302035, A2302004 to XC and YC), the National Natural Science 23 Foundation of China (Nos. 82430074, 82472294 to XC and YC), ……”. The correct **Funding** statement appears below.

The author(s) declared that financial support was received for this work and/or its publication. This work was supported by grants from the Shenzhen Medical Research Funding Program (Nos. B2302035, A2304001 to XC and YC), the National Natural Science Foundation of China (Nos. 82430074, 82472294 to XC and YC), the Science and Technology Innovation Commission of Shenzhen (Nos. JCYJ20220818095610021, JSGG20220822095200001 to YC), the Natural Science Foundation of Jiangxi Province (No. 20212BAB206077 to RH), the Science and Technology Project of Guangdong Province (No. 2025A1515010675 to YD), the Science and Technology Research Project of the Education Department of Jiangxi Province (No. GJJ2501322 to FL), and the Ganzhou Key Research and Development Project (No.GZ2024YLJ118 to JH). The funders had no role in the study design, data collection and analysis, decision to publish, or preparation of the manuscript.

The authors apologize for this error and state that this does not change the scientific conclusions of the article in any way.

The original article has been updated.

