# Supplementary material for: Distribution of granzyme-expressing NK cells in tuberculosis reflects subset and compartment-specific remodeling
Source: Front Immunol. 2026 Mar 17;17:1747972. doi: 10.3389/fimmu.2026.1747972 (PMC13035492; doi:10.3389/fimmu.2026.1747972)
Supplement: Supplementary file 1 [file Table1.docx]

**Supplemental material**

**Distribution of granzyme-expressing NK cells in tuberculosis reflects subset and compartment-specific remodeling**

**Authors:** Fuxiang Li^1,2^, Youchao Dai^3^, Shuixiang Xie^4^, Rong Hu^1,2^, Xueyun Gao^5^, Xiao Huang^2^, Shuxi Zhong^2^, Yi Cai^6^, Xinchun Chen^6#^, Junyun Huang^1,2#^

**Supplemental material**


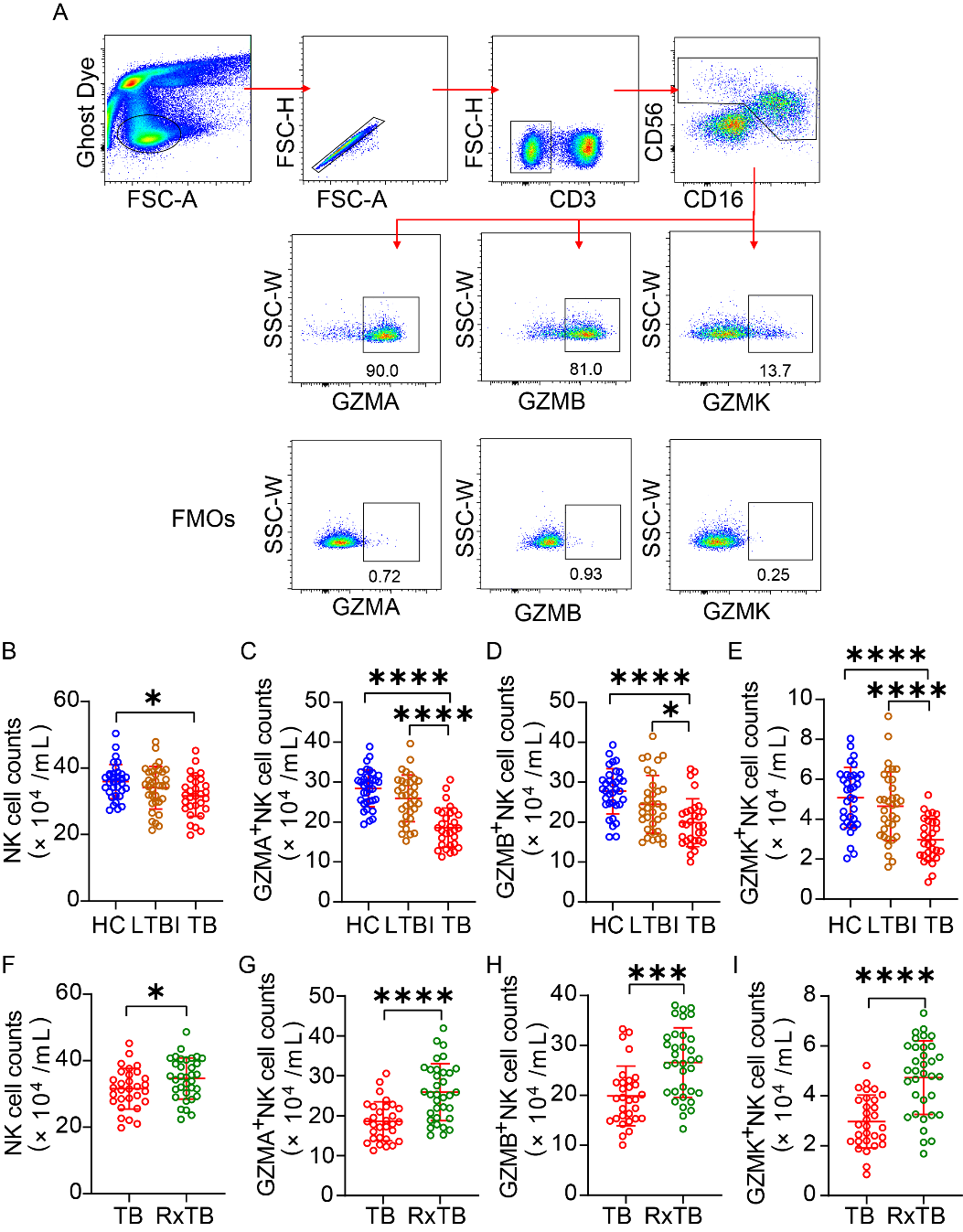


**Fig S1. Absolute counts of GZMA⁺/GZMB⁺/GZMK⁺ NK cells decrease during TB progression. (A)** Gating strategy for detecting granzyme-expressing NK cell subsets. Live lymphocytes were selected using Ghost Dye™ viability staining, then gated on CD3^–^CD56^+^ and CD3^–^CD16^+^ populations to define NK cells. Representative flow cytometry plots show identification of GZMA^+^, GZMB^+^, and GZMK^+^ subpopulations. **(B-E)** Absolute counts of total NK cells and distinct granzyme-positive subsets in peripheral blood from HCs, LTBI, and TB patients. **(F-I)** Absolute counts of total NK cells and distinct granzyme-positive subsets in peripheral blood from TB patients and RxTB individuals. Data are shown as mean ± SEM. **P* < 0.05, ****P* < 0.001, *****P* < 0.0001 by one-way ANOVA with Tukey’s multiple comparisons test (B–E) or unpaired Student’s t-test (F–I).


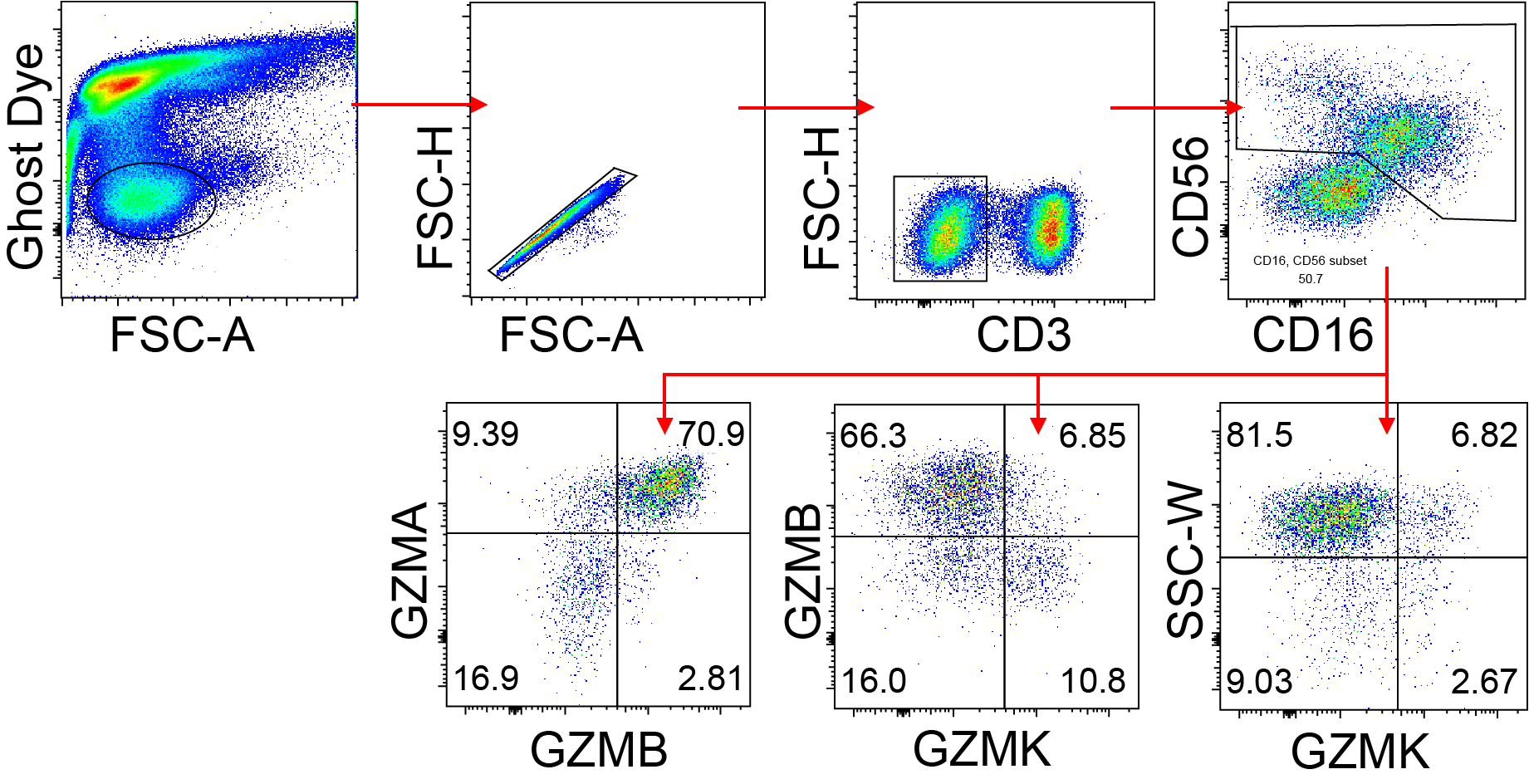


**Fig S2.** **Quadrant gating scheme for granzyme co-expression.** Following identification of live NK cells (Ghost Dye™^–^, CD3^–^, CD56^+^ or CD16^+^), bivariate plots were analyzed by quadrant gating to determine the frequency of cells single- or double-positive for the indicated granzyme pairs: GZMA/GZMB, GZMA/GZMK, and GZMB/GZMK.


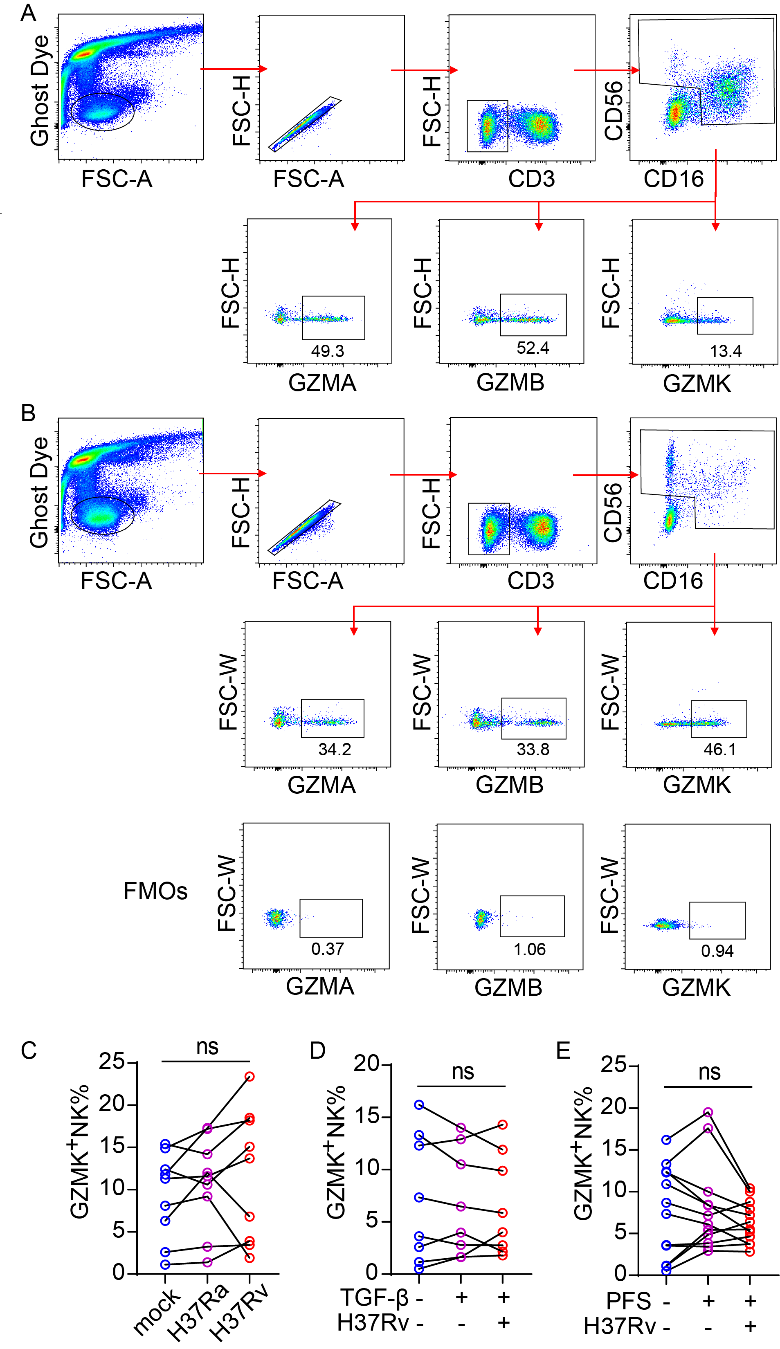


**Fig S3. Gating strategy for the analysis of granzyme-expressing NK cells in paired PBMC and PFMC samples. (A, B)** Gating strategy applied to representative samples from PBMC (A) and PFMC (B) to identify granzyme-expressing subsets within NK cells. Live lymphocytes were first selected using Ghost Dye™ viability staining, followed by gating on CD3^–^CD56^+^ and CD3^–^CD16^+^ populations to define NK cells. Subsequent plots show the identification of GZMA^+^, GZMB^+^, and GZMK^+^ subpopulations using quadrant gates. **(C)** GZMK^+^ NK cell induction in healthy donor PBMCs (n=9) after 48 h stimulation with medium (unstimulated), attenuated H37Ra (MOI=5), or virulent H37Rv (MOI=5). **(D, E)** Effects of pleural factors on GZMK^+^ NK frequency in healthy donor PBMCs (n=8) after 48 h stimulation with medium, TGF-β (10 ng/mL), TGF-β and H37Rv (MOI=5), 20% pleural fluid supernatant (PFS), or PFS+H37Rv (MOI=5). Data are shown as means ± SEM, ns, not significant, by one-way ANOVA with Tukey’s multiple comparisons test (C).


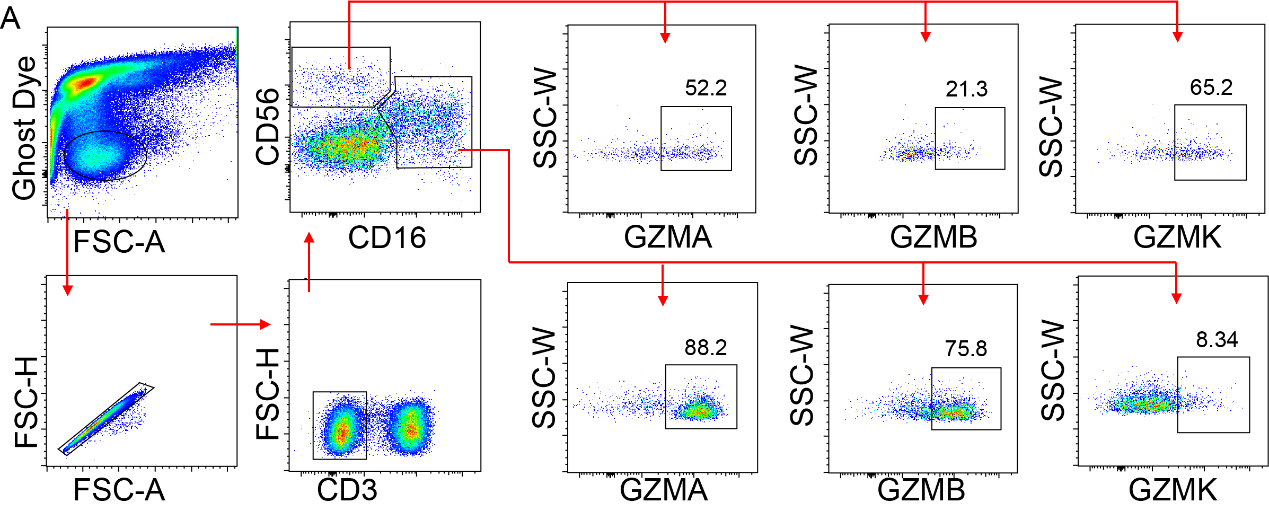


**Fig S4. Gating strategy for the analysis of granzyme expression in CD56^bright^ and CD56^dim^ NK cell subsets.** The methodology for assessing granzyme A, B, and K levels in CD56^bright^ and CD56^dim^ NK cells is shown. Following the identification of live, CD3^–^ cells, the population was split based on CD56 and CD16 expression level. Representative plots for GZMA, GZMB, and GZMK show the gating strategy applied to both subsets in parallel.


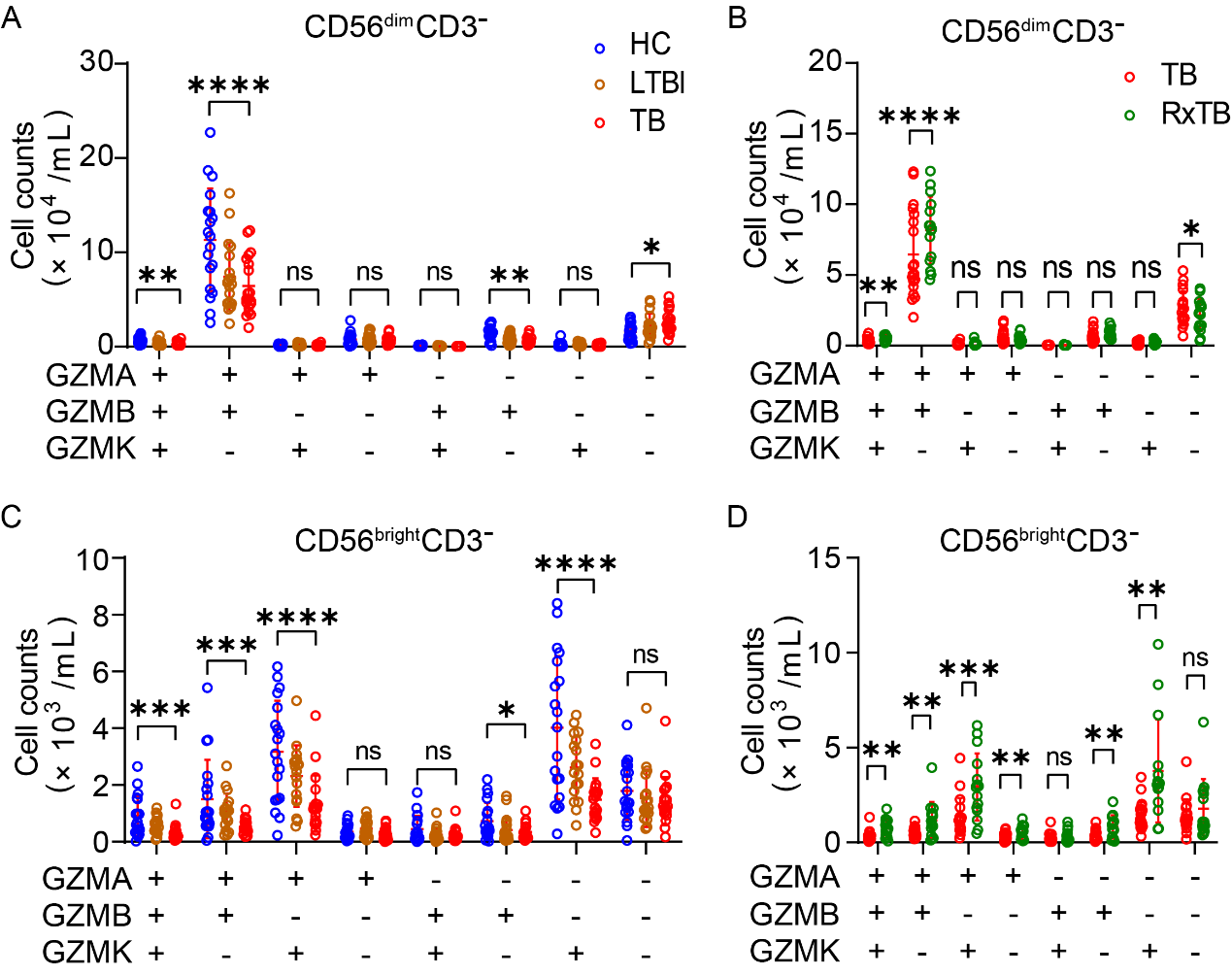


**Fig S5. Absolute counts of granzyme-co-expressing cells within CD56dim and CD56bright NK subsets**. (A, B) Absolute count of triple-granzyme co-expressing cells within the CD56dim NK subset. (C, D) Absolute count of triple-granzyme co-expressing cells within the CD56bright NK subset. Each dot represents an individual donor. Data are shown as mean ± SEM, ns, not significant, **P* < 0.05, ***P* < 0.01, ****P* < 0.01, and *****P* < 0.0001 by one-way ANOVA with Tukey’s multiple comparisons test (A, C) or unpaired Student’s t-test (B, D).


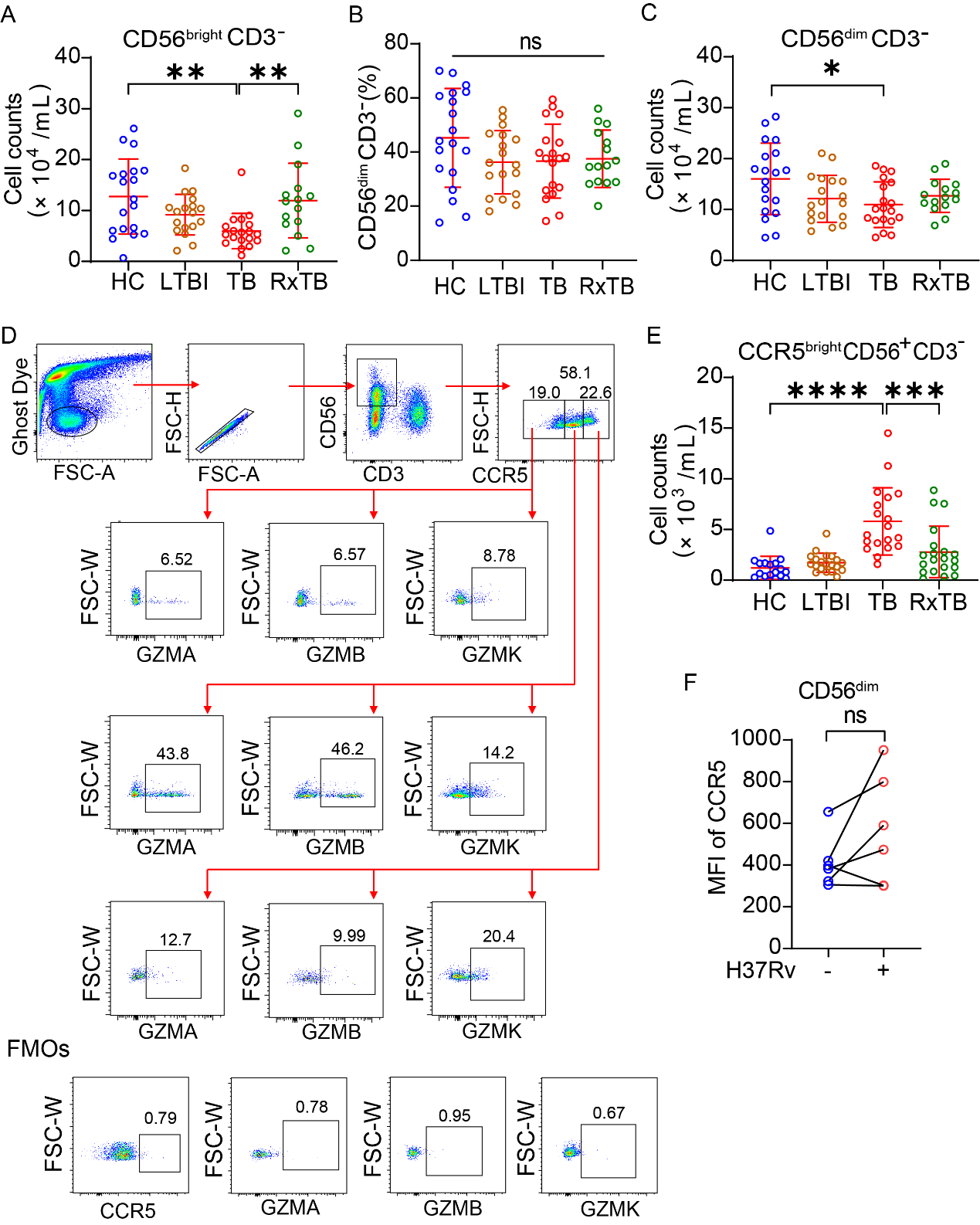


**Fig S6. Gating strategy for the analysis of CCR5-defined NK cell subsets and their granzyme expression. (A)** Absolute counts of CD56^bright^CD3^–^ in PBMCs from HCs, LTBI, TB, and RxTB. **(B, C)** Frequencies and absolute counts of CD56^dim^ NK cell subsets in PBMCs from HCs, LTBI, TB, and RxTB. **(D)** Representative gating strategy for identifying granzyme-expressing cells within CCR5-defined NK cell subsets from TB patient. After gating on live (Ghost Dye™^–^) lymphocytes and CD3^–^CD56^+^ NK cells, subsets were defined by CCR5 expression intensity (CCR5^–^, CCR5^dim^, CCR5^bright^). GZMA, GZMB, and GZMK expression was then assessed within each pre-gated CCR5 subset. **(E)** Absolute count of CCR5^bright^CD56⁺CD3^–^ NK cells in PBMCs from HCs, LTBI, TB, and RxTB. **(F)** Following *ex vivo* H37Rv infection (MOI = 5, 24 h), mean fluorescence intensity (MFI) of CCR5 unchanged on CD56^dim^ subsets. Data are shown as means ± SEM, ns, not significant, **P* < 0.05, ***P* < 0.01, ****P* < 0.01, and *****P* < 0.0001 by one-way ANOVA with Tukey’s multiple comparisons test (A-C, E) or unpaired Student’s t-test (F).

Supplemental table 1 Demographic characteristics of study populations

| Subject | Case No. | Sex (M/F) | Age (range) | AFB-positive | Elispot Positive |
| --- | --- | --- | --- | --- | --- |
| HC | 37 | 17/20 | 36.2 (20-55) | ND | 0 |
| LTBI | 40 | 19/21 | 40.2 (26-58) | ND | 40 |
| TB | 36 | 13/23 | 37.7 (19-60) | 36 | 36 |
| RxTB | 34 | 15/19 | 38.2 (21-57) | 34 | 34 |
| TPE | 22 | 16/6 | 35.1 (25-56) | 22 | 22 |
